# Workgroup Report: Review of Genomics Data Based on Experience with Mock Submissions—View of the CDER Pharmacology Toxicology Nonclinical Pharmacogenomics Subcommittee

**DOI:** 10.1289/ehp.8318

**Published:** 2005-11-03

**Authors:** John K. Leighton, Paul Brown, Amy Ellis, Patricia Harlow, Wafa Harrouk, P. Scott Pine, Timothy Robison, Lilliam Rosario, Karol Thompson

**Affiliations:** 1 Office of New Drugs and; 2 Office of Pharmaceutical Science, Center for Drug Evaluation and Research, U.S. Food and Drug Administration, Silver Spring, Maryland, USA

**Keywords:** data visualization, electronic data files, MIAME/MINTox, mock submission content, quality control metrics, toxicogenomics

## Abstract

Over the past few years, both the U.S. Food and Drug Administration (FDA) and the pharmaceutical industry have recognized the potential importance of pharmacogenomics and toxicogenomics to drug development. To resolve the uncertainties surrounding the use of microarray technology and the presentation of genomics data for regulatory purposes, several pharmaceutical companies and genomics technology providers have provided the FDA with reports of genomics studies that included supporting toxicology data (e.g., serum chemistry, histopathology). These studies were not associated with any active drug application and were exploratory or hypothesis generating in nature. For training purposes, these reports were reviewed by the Nonclinical Pharmacogenomics Subcommittee consisting of the Center for Drug Evaluation and Research pharmacology and toxicology researchers and reviewers. In this article, we describe some of these submissions and report on our assessment of data content, format, and quality control metrics that were useful for evaluating these nonclinical genomics submissions, specifically in relation to the proposed MIAME/MINTox (minimum information about a microarray experiment/minimum information needed for a toxicology experiment) recommendations. These genomics submissions allowed both researchers and regulators to gain experience in the process of reviewing and analyzing toxicogenomics data. The experience will allow development of recommendations for the submission and review of these data as the state of the science evolves.

To share and gain expertise in toxicogenomics and to develop a framework for reviewing data, pharmacology and toxicology reviewers and researchers within the U.S. Food and Drug Administration (FDA) Center for Drug Evaluation and Research (CDER) formed the Nonclinical Pharmacogenomics Subcommittee (NPSC) (CDER 2004). The NPSC has reviewed data from several “mock submissions” of nonclinical studies that were voluntarily submitted to the committee or to review divisions. The term “mock submissions” is used in this report as opposed to “voluntary submissions’’ discussed in the FDA pharmacogenomics data submissions guidance (FDA 2005b), which are to be reviewed by the FDA Interdisciplinary Pharmacogenomics Review Group (CDER 2005a, 2005b). From this work, the NPSC gained insight into toxicogenomics data submissions and identified areas that might need additional consideration before full integration of this technology into routine drug development. In this article we summarize the most useful portions and identify potentially unnecessary details in these mock submissions and provide insight as to how CDER reviewers assess submissions of pharmacogenomics data.

## General Description of Mock Submissions

The NPSC examined in detail three mock submissions that addressed different uses of pharmacogenomics data during drug development. The topics addressed by the mock submissions were *a*) microarray data quality using a data set generated for a single compound, *b*) assessment in a single tissue of the effects of several compounds in a pharmacological class, and *c*) use of a proprietary database to help classify a compound of unknown toxicity. None of the submissions involves a compound that is the subject of an active investigational new drug (IND) or new drug application (NDA). Further, these studies were not conducted under Good Laboratory Practice (GLP) regulations for nonclinical studies regulations (FDA 2002), and deviations from GLP were not described in the submissions. The NPSC did not judge the scientific merit of the conclusions reached by the sponsor of each mock submission but rather considered the experimental design, organization of data, and adequacy of the information available to support the hypothesis, including the relationship of the genomics data to the general toxicology data.

The NPSC evaluated the content of the mock submissions using the framework for reporting microarray data outlined by the Microarray Gene Expression Data (MGED) Society in their proposals of the minimum information about a microarray experiment (MIAME) (Brazma et al. 2001; MGED Society 2002). The fields specified in MIAME present a foundation for content necessary for review of pharmacogenomics data. More recently, a framework for reporting biological investigations across “omics” technologies has been initiated through the MGED Society that includes an investigational design description checklist, a discipline-specific checklist [e.g., MINTox (minimum information needed for a toxicology experiment)], and a technology-based checklist (e.g., MIAME) (MGED Society 2004). The mock submissions were not specifically prepared according to the MIAME/MINTox recommendations; however, one submission was prepared to conform to the specifications of the draft pharmacogenomics guidance (FDA 2003).

We describe in detail the three submissions in the sections that follow, with the sponsor’s conclusions and the NPSC comments for each submission. Table 1 summarizes the formats for various parameters used in each submission.

## Submission 1: Assessment of Data Quality for Microarray Studies on a Single Agent

### Submission description.

The mechanism of action of a compound purported to reduce cholesterol levels by inhibiting HMG-CoA (3-hydroxy-3-methylglutaryl coenzyme A) reductase was investigated in female rats. The compound was administered daily by oral gavage at three dose levels for 1 month. Observations and measurements included clinical assessments (including body weights and food consumption), serum chemistry, macroscopic and microscopic evaluations of kidney and liver, and quantitative polymerase chain reaction (qPCR) of HMG-CoA reductase and cytochrome P450 (*CYP*)*1B1* and *CYP2B2* gene expression in rat liver. The report included all the data except for the histopathological findings in kidney and liver. Detailed protocols for microarray assay steps from RNA isolation to array scanning were provided in the submission. Gene expression analysis of liver samples collected 1, 7, or 30 days after drug administration was performed using Affymetrix (Santa Clara, CA) Rat Genome U34 Microarrays. Only the concurrent control and high-dose groups were analyzed for gene expression changes.

The submission addressed the potential use of controls to monitor different steps in microarray experiments. Although labeling controls to monitor the efficiency of target preparation were not included in the mock data set, the sponsor included proprietary data on the use of polyadenylated transcripts of three *Bacillus subtilis* genes (*lys*, *phe*, and *thr*; GenBank accession nos. X17013, M24537, X04603, respectively; http://www.ncbi.nih.gov/GenBank) to illustrate how this information might be incorporated in an actual microarray data submission. Hybridization controls to determine the sensitivity and quality of hybridizations included the measurement of *BioB*, *BioC*, and *BioD* genes (GeneBank accession no. J04423; http://www.ncbi.nih.gov/GenBank/) of the *Escherichia coli* biotin synthesis pathway and the *cre* recombinase gene for P1 bacteriophage (GeneBank accession no. X03453; http://www.ncbi.nih.gov/GenBank/). Data from these hybridization controls were provided in tabular and graphic form, including an analysis of the dynamic range. The sponsor also discussed the possible use of batch controls to monitor technical variability and equipment controls to ensure microarray image quality.

The submission included a comprehensive section on quality control (QC) metrics. The sponsor provided examples based on their general laboratory experience to demonstrate the possible use of QC metrics for RNA and cRNA because these metrics were unavailable for the submitted data set. RNA quality was assessed by 28S:18S ratios and percent area (defined as the percentage of the total RNA population that is derived from the 18S and 28S rRNA bands). For cRNA, *in vitro* transcription yield and the ratio of absorbance at 260 and 280 nm were proposed as QC metrics. QC metrics generated by Affymetrix Microarray Suite 5.0 (MAS5: Affymetrix) software from scanned images (i.e., background, noise, scaling factor, percent absent, 3′:5′ ratios) were used to assess the quality of the hybridizations.

These metrics informed a paradigm for rejection of array data. Further, hybridization images were inspected for signal level, presence of image defects, and appropriate grid alignment at the edges and corners. Reproducibility between biological replicate hybridizations (correlation, detection call agreement, and < 2-fold signal value difference) was presented as a scatter plot and in tabular form. The bias in the data due to scanning of control and treated samples on different days was identified through examination of the data for the hybridization controls. The bias was mitigated by using a permutation analysis to reduce the number of false positives and by considering the biological relevance of the differentially expressed genes.

### Sponsor’s conclusions.

Analysis of changes in liver gene expression was conducted to further understand the pharmacological mechanism of action of the compound through examination of its primary genomics target in the cholesterol biosynthesis pathway. The compound showed no significant treatment-related toxicity in the liver at the tested doses. A heat map was generated to compare samples collected from treated and control animals at different time points after drug administration. The heat map illustrated possible co-regulated genes that clustered by direction and extent of regulation at each time point. Changes in expression levels of genes encoding enzymes from relevant biochemical pathways were shown in figures and discussed in the text. The data were consistent with current understanding of cholesterol biosynthesis.

### NPSC comments.

The content and organization of the mock submission were a collaborative exercise between the NPSC and the sponsor. A preliminary technical plan was presented to the NPSC and followed by a draft proposal before the final mock submission. The sponsor submitted very extensive information on laboratory and informatics infrastructure. Such extensive information may not need to be included in all genomics data submissions where a summary is generally sufficient. However, this information should be well documented and available on request, possibly as part of a device master file or standard operating procedures (SOPs). Although the text of the submission contained information allowing a reviewer to trace a sample from the animal source to its corresponding array data set, it would have been helpful for the sponsor to provide a key in electronic file format to link these files.

The sponsor submitted a comprehensive section on QC measurements. The NPSC concluded that these parameters appeared adequate. The electronic files were organized to be consistent with FDA guidance (FDA 1999). The NPSC noted the importance of electronic data sets, even for subsets of genes. The utility and suitability of particular electronic formats will depend on whether these files are used to populate a database with data-mining capabilities or as a data repository. In the review of electronic microarray data, the NPSC noted the need for user-friendly software tools to analyze and visualize data. It was apparent that reviewers will need appropriate training and software to effectively manipulate microarray-associated data.

The NPSC observed that, in general, the protocols for the in-life portion of toxicogenomics studies should follow practices used for standard toxicity studies. These include the use of both sexes (unless there is scientific justification to limit the study to one sex) and using appropriate doses of drug. The number of replicates and power needed will vary depending upon the sponsor’s intended claim and the experimental objectives (Simon et al. 2002). However, in order to perform some minimal statistical analysis, at least three animals per sex per time point are generally needed. Often, it may be necessary to use doses of drug large enough to induce the toxicity or pharmacologic activity that is related to the genomics target being studied. Gene expression changes that are critical to the sponsor’s argument may be confirmed with qPCR on a limited number of genes. The NPSC believed that this strengthened the experimental data in this submission, considering the limited experience with gene expression data and the continuing evolution of the technology. Differences between sample collection methods (transverse vs. longitudinal sectioning) for histopathology and genomics analysis were not explained and could be considered a complicating factor in an analysis. The NPSC also agreed that it would facilitate review if the animal data portions of toxicogenomics studies were submitted in the format of a standard GLP toxicology study.

In general, the NPSC agreed that sufficient data were available to support the mechanism of compound action proposed by the sponsor. The NPSC considered the use of clustering analysis to identify co-regulated genes to be primarily hypothesis generating. Pharmacogenomics data that explore the presumptive mechanism of action of a compound may enhance traditional toxicology studies.

## Submission 2: Assessment in a Single Tissue of the Effects of Several Compounds in a Class

### Description.

The potential human liver toxicity of compounds used to treat dyslipidemia was evaluated by treating male cynomolgus monkeys with one of three drugs. The test articles included two marketed peroxisome proliferator α-agonists (PPARα; fenofibrate and ciprofibrate) and a proprietary PPAR pan-agonist compound (PPARpan) with activity on PPARα, γ, and δ receptors. A vehicle control was also included. In the definitive study, monkeys (four per group) were treated for 15 days with a single dose of PPARpan or several dose levels for fenofibrate or ciprofibrate, respectively. Doses for fenofibrate and ciprofibrate in the 15-day study were based on a 4-day dose-range–finding study. *In vivo* study observations included clinical evaluations (including body weight and food consumption), clinical chemistry and hematology, and toxicokinetics. At necropsy, organ weights were determined and macroscopic evaluation conducted. The sponsor used SOPs in conducting the toxicology portion of the study. The livers were examined using both light microscopy and transmission electron microscopy (TEM). Enzymatic activity of components of β-oxidation pathways was also assessed in these samples.

Liver sections were collected for toxicogenomics analysis from all animals. RNA was isolated and the expression profile was examined using the manufacturer’s standard protocols with Affymetrix GeneChip HGU95Av2 arrays. The mock submission provided limited information about the protocols because these protocols are publicly available. Samples reported included control and high-dose animals from the range-finding study and all animals in the definitive study.

### Sponsor’s conclusions.

To further understand the toxicity associated with PPARpan, the sponsor analyzed microarray data using principal component analysis (PCA) to examine the relationship between control and treatment groups in both the 4-day dose-range–finding study and the 15-day definitive study. The samples from animals treated for 4 and 15 days clustered differently. The sponsor attributed these differences to technical factors based on a similar separation among 4-and 15-day control animals. One 15-day control animal clustered away from other samples in the same treatment group and thus appeared to be an outlier. Ciprofibrate-treated samples clustered more tightly by dose level than fenofibrate-treated samples and demonstrated more pronounced dose–response alterations in gene expression. The single-dose level of PPARpan induced a modest alteration in gene expression. Using PCA, PPARpan effects appeared similar to those of the higher dose levels of fenofibrate.

The sponsor assumed that the human-based probe sets with a MAS5 detection call of “present” were hybridizing to the correct homologous monkey sequence and that the data were interpretable because the objective was to compare gene expression between control and treated groups. Previous studies have demonstrated that cRNA from macaque and rhesus monkeys effectively hybridizes with a human Affymetrix GeneChip (Chismar et al. 2002; Enard et al. 2002), although a recent study (Gilad et al. 2005) found a large effect of sequence divergence on hybridization signal. The sponsor concluded that some of the interanimal variability seen in this study may be due to greater genetic heterogeneity of monkeys compared with laboratory rodents and to imperfect hybridization of monkey cRNA to the human array.

Treatment-related effects were noted in clinical observations and serum chemistry, particularly at the highest doses. Liver changes reported at some or all drug-treatment groups included hypertrophy, increased liver weights, eosinophilia and granularity of the cytoplasm, and single-cell necrosis consistent with apoptotic cell death. This last finding was not observed with PPARpan. TEM evaluation indicated that all compounds increased peroxisome and mitochondria number as well as mitochondrial area. Activity of some enzymes of β-oxidation was increased in livers of ciprofibrate- and fenofibrate-treated animals. Further interpretation of the gene expression results, for example, regarding induction of apoptosis, has subsequently been performed by the sponsor (Cariello et al. 2005).

### NPSC comments.

The initial submission consisted of separate toxicology and gene expression study reports of the same experiments. Although some experimental data such as animal treatment and husbandry were available through cross-reference to other protocols, this submission would have been easier to review if it had been organized as an integrated report using a format like MIAME/MINTox. Data for QC metrics were provided in the submission, but graphical presentation of some metrics would have aided the review. The toxicology data were very helpful in interpreting the difference in gene expression results between the PPARα compounds and PPARpan. The electron microscopy and morphometry analysis of peroxisomes and mitochondria provided additional detail relevant to the toxicogenomics assessment.

Inclusion of electronic files in the mock submission made it possible to independently analyze the sponsor’s results. The NPSC used Spotfire software (Spotfire Inc., Somerville, MA) to analyze the microarray data from the 4- and 15-day experiments. The NPSC chose not to analyze these data sets together because the significant technical variation between them might confound the analysis of biological relatedness between PPARpan and PPARα agonists. Cariello et al. (2005) noted that poor-quality RNA was obtained from one control animal and that the arrays from the 4-day non-GLP study and the 15-day GLP study were processed on different days by different individuals. However, the arrays for the controls and treated groups for each day were processed together. Therefore, the results for the treated groups can be compared separately with the results for the appropriate control.

**Figure 1:**
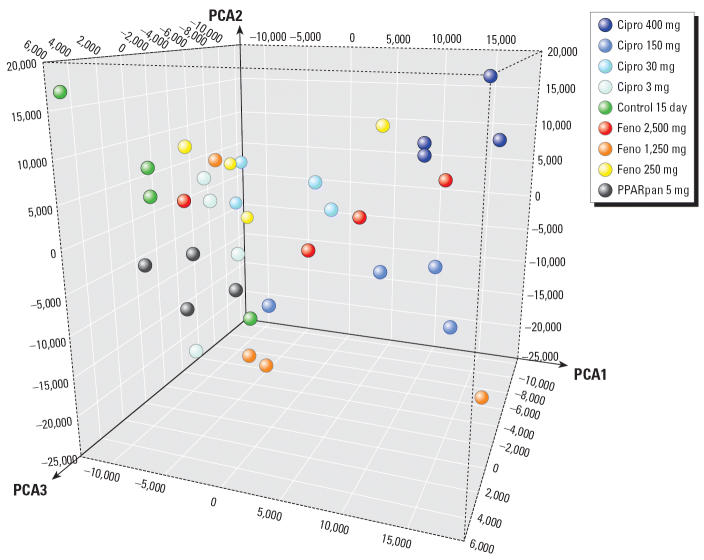
PCA of gene expression signals (expressed as arbitrary units) in cynomolgus monkey liver samples after 15-day treatment with hyperlipidemia drugs. Cipro, ciprofibrate; Feno, fenofibrate.

PCA by the NPSC of individual samples from the 15-day experiment showed that most samples did not form tight clusters within their treatment groups (Figure 1). This indicated that gene changes at the low dose were as likely to be related to biological and technical variation as to drug treatment. In contrast, a clear dose-related separation was observed for the ciprofibrate treatment. In this analysis, PPARpan samples clustered in the same region of the PCA as the control and low-dose treatment samples. This differed from the sponsor’s conclusions, which were derived from an analysis using both 4- and 15-day treatment groups. Additionally, the NPSC concluded that the control animal in the 15-day experiment identified as an outlier by the sponsor did not appear to be more distant from other control samples in the PCA when compared with other dose group distance variations. Although PCA is a valuable method to inspect sample homogeneity and general treatment-related changes in gene expression data, it is a subjective and relative method of analysis. Additional analyses of the data (e.g., using heat maps, pathway analysis) would have been useful to provide more support for the conclusions. These analyses have subsequently been performed and published by the sponsors (Cariello et al. 2005).

Although cross-species hybridization is not an ideal approach, there are limitations in the available arrays for specific animal models. The sponsor acknowledged this fact and attempted to address it by providing confirmatory evidence with additional studies. With the advent of arrays for additional laboratory species, including the monkey, this technical challenge is not expected to be a major regulatory concern in the future.

Despite the technical problems encountered and the different conclusions reached by the sponsor and the NPSC, this submission provided an example of the incorporation of gene expression analysis into an overall assessment of the potential toxicity of a new drug entity compared with a known drug class. Such an integrated submission potentially could be used to support a possible mechanism of action or to distinguish compounds within a pharmacologic class.

## Submission 3: Assessment of Toxicity of a Test Compound by Comparison with a Reference Database

### Description.

The potential toxicity of a selective serotonin reuptake inhibitor (SSRI) was assessed in male rats treated daily for 5 days with vehicle and two dose levels of a test compound. One dose was a nontoxic pharmacologic dose, whereas the high dose was equivalent to maximum tolerated dose based on decreases in body weight gain. The results of molecular pharmacology assays, clinical observations (including body weights and food consumption), clinical chemistry and hematology, selected organ weights, necropsy observations, and liver histopathology were provided for four time points (6 hr to 6 days after the first dose was administered). Liver samples from individual animals were obtained and processed separately for microarray analysis. Gene expression studies were conducted using CodeLink RU1 Expression BioArrays (Amersham Biosciences, Piscataway, NJ). Expression data for three to six animals per time point per treatment group dosed for 1, 3, and 5 days were analyzed and compared with a contextual reference database of microarray data for approximately 600 compounds. The sponsor provided access to its proprietary database to allow NPSC members to independently confirm the gene expression findings.

### Sponsor’s conclusions.

The toxicogenomics analysis indicated that the SSRI was relatively nonhepatotoxic, as confirmed by the histopathology findings. The sponsor defined a drug signature as “a small set of genes that delineates a property of one class of compounds from another or from vehicle controls.” The use of drug signature analysis confirmed several effects of the SSRI class that were observed using traditional pharmacology and toxicology assays (ion channel blocking and serum creatinine increase). Signature analysis suggested potential safety risks of perturbed blood pressure regulation and phospholipidosis, but ancillary data were not available to confirm these findings.

### NPSC comments.

This mock submission demonstrated the use of a contextual database containing genomics data annotated with toxicity data for the interpretation of the potential toxicity of an SSRI. The comparison of the pharmacology data and gene signature profile for this SSRI with similar compounds in the database also provided an example of gene profile specificity. In this example, independent verification of matches to some gene signatures was provided by clinical chemistry and molecular pharmacology assay results. Matches to other gene signatures could only suggest potential toxicities, as these are not probable valid biomarkers. The signatures would require additional experiments for confirmation. Potential toxicities suggested by gene signatures would be addressed in different sections of full IND/NDA submissions (e.g., safety pharmacology and histopathology in a longer toxicology study). The submission contained adequate information on sample and array quality assessment and on the statistical analyses that were applied to the data. The NPSC agreed that sufficient data were available to support the hypothesis proposed by the sponsor.

The opportunity for the NPSC to access and be trained on the database provided an in-depth understanding of the use of a contextual database to generate gene expression signatures. Voluntary submissions may be a suitable route for sponsors to introduce FDA reviewers to similar approaches that use large data sets to generate gene signatures. The NPSC anticipates increased use of contextual databases as a resource for the generation of gene expression signatures or motifs that could eventually become validated biomarkers of toxicity. However, the gene components of these signatures would need to be available for review by regulators if not generally accepted, that is, a known valid biomarker, as defined by the FDA pharmacogenomics guidance (FDA 2005b).

## Other Submissions Containing Pharmacogenomics Data

The NPSC is aware of several nonclinical pharmacogenomics submissions provided as part of IND and NDA submissions. These gene expression studies were designed to explore the pharmacology of the pharmaceutical or as an additional tool to investigate an observed toxicity. As described by the FDA pharmacogenomics guidance (FDA 2005b), most of these submissions likely would have qualified as voluntary submissions to the IND or as synopses to the NDA because they were exploratory studies in which no regulatory decisions were or could have been made. The sponsors were not using the pharmacogenomics data to make claims regarding safety or efficacy. The submissions did not involve either known or probable valid biomarkers. MIAME/MINTox criteria were not used in these submissions. All submissions were provided as paper reports, making it difficult to assess drug-related effects on gene expression. Most of these submissions reported only a subset of affected genes, which may be acceptable, although all data should be available upon request. In the opinion of the NPSC, submission of the data in an electronic format would have facilitated review of these analyses. Some of these studies were inadequately designed. Issues included insufficient numbers of animals per group per time point, too few time points, use of pooled tissue samples, and lack of QC metrics (e.g., demonstration of RNA purity). Adequate documentation and evaluation of these parameters will be important for future regulatory submissions.

## Conclusions

The application of pharmacogenomics and toxicogenomics in drug development has primarily been used in compound selection and for identification of possible biomarkers of safety or efficacy. The NPSC anticipates that as confidence grows in the technology and guidelines for its use in a regulatory context are further delineated, sponsors will increasingly use it to address issues of regulatory importance. These may include studies of a drug’s mechanism of action or further investigation of a specific toxicity observed in a clinical or non-clinical study. The mock submissions described here serve as a basis for dialog within and outside the FDA to address how data are to be submitted, what data should be submitted, and what regulatory decisions are likely to be made with the data submitted.

A structure such as that described by MIAME/MINTox (MGED Society 2004) would be useful for review of genomics data within the context of a drug approval submission. MIAME/MINTox is a checklist of information important for independent review of genomics data within a biological context. Genomics data submissions were easier to review when integrated into a standard toxicology report format. It would be useful to include pathway analysis and other gene annotations with lists of gene changes. Confirmation of gene changes by secondary analysis (e.g., PCR) may be included to support conclusions drawn from the genomics expression analysis. The need for such confirmation may depend on the sponsor’s claim and its impact on the safe use of the drug being tested. If genomics data were part of a standard IND/NDA submission, any additional toxicity suggested by these data would be addressed in the standard safety pharmacology studies and in longer toxicology studies typically performed during drug development.

Analyses such as PCA were helpful for identifying general similarities or differences among samples within or across treatment groups. Information not normally included in most submissions of toxicology data and not specified in MIAME/MINTox, such as information on laboratory informatics and equipment settings, may not be needed for review but should be available upon request. For example, information on array design description for commercially available arrays may not be necessary, but specifications should be provided for custom arrays. Quality metrics that were used for technical evaluation of the microarrays in these submissions were generally acceptable, but additional standards may be necessary for use of gene expression analysis in nonclinical toxicology assessments during drug development. It is not clear how much information regarding technical variation and equipment efficiency will be needed in a regulatory submission.

The mock submissions were a useful tool for the NPSC to gain experience in how to best review toxicogenomics data. Additional voluntary genomics data submissions as described in the pharmacogenomics guidance (FDA 2005b) are encouraged so that the best practices for handling these data can continue to be developed. Many issues remain to be addressed. These include but are not limited to the amount of data needed to support a study’s conclusion, methods for the statistical evaluation of microarray data, the complexity of pathway analysis, and the need to make decisions concurrently with advances in related disciplines such as cell biology and molecular medicine.

The FDA has co-hosted several workshops on pharmacogenomics and drug development with groups representing pharmaceutical manufacturers and the biological industry. Additional workshops are planned in the near future. Consortia have been formed to continue dialogue between regulators, industry groups, and academicians on these topics under the umbrella of organizations such as the International Life Sciences Institute (ILSI) through the ILSI–Health and Environmental Sciences Institute initiative (Pennie et al. 2004). The Clinical Data Interchange Standards Consortium nonclinical working group is developing hypothetical case examples to address specific toxicity issues, with the goal of enhancing the development and acceptance of toxicogenomics data standards (Lord and Papoian 2004). In time, this ongoing dialogue and additional opportunities to review genomics data promise to lead to a more rapid development program for novel pharmaceuticals, as envisioned by the FDA’s Critical Path Initiative (FDA 2004).

## Figures and Tables

**Table 1:** Format of content in the mock submissions.

Parameter	Submission 1	Submission 2	Submission 3
Study design	Supplied in both MS Word and PDF formats	In PDF file	In MS Word document
Toxicology	In SAS transport files	Complete reports, including TEM, in PDF files	In MS Word document; molecular pharmacology results also provided
Microarray platform	Affymetrix GeneChip (rat genome U34 arrays)	Affymetrix GeneChip (human HGU95Av2 arrays)	CodeLink RU1 Expression BioArrays (rat)
Microarray protocol information	Affymetrix EXP files	Not provided	In MS Word document
QC metrics	Affymetrix RPT files and RNA QC metrics (see text)	Results from Affymetrix RPT files combined in a single TXT file	Agilent Bioanalyzer electropherograms, QC metrics for RNA and arrays, and correlation to historic control data provided in MS PowerPoint and MS Excel files
Raw expression data	Array images as low-resolution figures in Word document	Not provided	Array images provided as TIFFs
Processed expression data	Provided in XML, CHP, CEL, and SAS transport files	MAS5 signals and detection calls	Provided in TXT files
Interpreted expression data	Subsets of significantly changes genes in MS Excel files; affected pathways in MS PowerPoint files	PCA results provided as bitmaps and interpreted in PDF file	Two-dimensional hierarchical clustering, Pearson’s correlation, linear discriminant signature analysis, pathway analysis
Statistical analysis	Affymetrix MAS5 using two different data normalization algorithms	Rosetta Resolver (version 3.2) (*p*-values, fold-change, and other parameters)	Tools in proprietary database
Notable inclusions	A detailed description of the statistical preprocessing of expression data, filtering, normalization, and modeling methods	A separate tab-delimited text file contained data for all probe sets with a *p*-value < 0.05	Contextual gene expression and drug signature analysis; electronic file tracking key

Abbreviations: EXP, experiment information file; MS, Microsoft; PDF, portable document format; RPT, report file; TEM, transmission electron microscopy; TIFF, tagged image file format; TXT, text file.
